# Comparison of commercially available differentiation media on cell morphology, function, and anti-viral responses in conditionally reprogrammed human bronchial epithelial cells

**DOI:** 10.1038/s41598-023-37828-0

**Published:** 2023-07-11

**Authors:** Nikhil T. Awatade, Andrew T. Reid, Kristy S. Nichol, Kurtis F. Budden, Punnam Chander Veerati, Prabuddha S. Pathinayake, Christopher L. Grainge, Philip M. Hansbro, Peter A. B. Wark

**Affiliations:** 1 School of Medicine and Public Health, University of Newcastle, Callaghan, NSW Australia; 2 Immune Health Program, Hunter Medical Research Institute, University of Newcastle, New Lambton Heights, NSW Australia; 3 Asthma and Breathing Research Program, Hunter Medical Research Institute University of Newcastle, New Lambton Heights, NSW Australia; 4 Centre for Inflammation, Centenary Institute and University of Technology Sydney, Sydney, NSW Australia; 5 Dept of Respiratory and Sleep Medicine, John Hunter Hospital, New Lambton Heights, NSW Australia

**Keywords:** Respiration, Molecular biology, Physiology

## Abstract

Primary air liquid interface (ALI) cultures of bronchial epithelial cells are used extensively to model airway responses. A recent advance is the development of conditional reprogramming that enhances proliferative capability. Several different media and protocols are utilized, yet even subtle differences may influence cellular responses. We compared the morphology and functional responses, including innate immune responses to rhinovirus infection in conditionally reprogrammed primary bronchial epithelial cells (pBECs) differentiated using two commonly used culture media. pBECs collected from healthy donors (n = 5) were CR using g-irradiated 3T3 fibroblasts and Rho Kinase inhibitor. CRpBECs were differentiated at ALI in either PneumaCult (PN-ALI) or bronchial epithelial growth medium (BEGM)-based differentiation media (BEBM:DMEM, 50:50, Lonza)—(AB-ALI) for 28 days. Transepithelial electrical resistance (TEER), immunofluorescence, histology, cilia activity, ion channel function, and expression of cell markers were analyzed. Viral RNA was assessed by RT-qPCR and anti-viral proteins quantified by LEGENDplex following Rhinovirus-A1b infection. CRpBECs differentiated in PneumaCult were smaller and had a lower TEER and cilia beat frequency compared to BEGM media. PneumaCult media cultures exhibited increased *FOXJ1* expression, more ciliated cells with a larger active area, increased intracellular mucins, and increased calcium-activated chloride channel current. However, there were no significant changes in viral RNA or host antiviral responses. There are distinct structural and functional differences in pBECs cultured in the two commonly used ALI differentiation media. Such factors need to be taken into consideration when designing CRpBECs ALI experiments for specific research questions.

## Introduction

Air liquid interface (ALI) cultures of primary bronchial epithelial cells (pBECs) have proven to be an invaluable tool for studying respiratory diseases, providing a human cell model that recapitulates features of the airway epithelium in vivo^1,2^. The major limiting factors are the paucity of pBECs which can be obtained and cultured ex vivo from endobronchial brushing, as primary cells have a finite capacity for expansion and proliferation^3,4^. Conditional reprogramming (CR) is an increasingly popular technique to increase the number of pBECs available for investigation. Here, the concomitant use of γ-irradiated feeder cells and rho kinase (ROCK) inhibitor have enabled large-scale expansion of pBECs, facilitating investigations of cell–cell interactions, morphology, epithelial cell function and interventions with limited starting cell numbers^4–6^.

While most laboratories have adopted a CR expansion method, several different media are available for the subsequent differentiation into ALI culture including Epi (Epithelix)^7^, EMM (PromoCell, Epithelix)^7^, mAir (PromoCell)^7^, bronchial epithelial growth medium (BEGM) based differentiation media (BEBM:DMEM, 50:50, Lonza)^8^ and PneumaCult ALI (STEMCELL Technologies)^9^. The constituents of most differentiation media are proprietary but are certainly distinct since the use of different differentiation media has been shown to affect stratification, cell phenotypes and the proportions of different cell types^10^. This can impact the consistency, reproducibility, and comparability of research findings. Several studies have examined differences between alternate epithelial cell differentiation media^7,11–14^, but, given the increasing prevalence of CR-expanded ALI cultures, it is now necessary to examine how these media may affect differentiation of CRpBECs, which, have not been investigated.

Of the few studies using CRpBECs, one used nasal epithelial cells and the other bronchial, though both assessed morphology, differentiation phenotype and assessed limited functional characteristics, leaving a gap in the understanding of the impact of differentiation media on these cells (studies are summarized in Table 1).

**Table 1:** Previous studies that compared different commercial differentiation media.

Publications	Expansion by CR	Differentiation media	Bronchial/ nasal/tracheal cultures	Thickness of cultures	Resistance	Cilia density	Cilia beat frequency	Ion channel function	Cell morphology (cell size)	Mucosecretory markers (MUC5AC, goblet cells)	Virus infectivity	Inflammatory chemokines
Current study	Yes (F media)	AB-ALI vs PN-ALI	Bronchial (adult)	Thicker in PN-ALI	Lower in PN-ALI	Higher density and FOXJ1 expression in PN-ALI	Higher in AB-ALI	Similar CFTR currents, higher CaCC currents	Smaller, more tightly packed cells in PN-ALI	Higher MUC5AC, but not significant in PN-ALI	More RSV infected cells in PN-ALI but not significant	Similar in both media
Livnat et al. ^14^	No	MEM USG-ALI ALI from UNC PN-ALI	CFBE41o Cells (bronchial) stably expressing cell line	–	–	–	–	Higher wt-CFTR currents in MEM media	–	–	–	–
Luengen et al. ^7^	No	mAir, PN-ALI, Epi, EMM	Nasal (donors between 10 and 40 years old)	–	Measured but not mentioned which media had better resistance	Increased ciliation in mAir media. Lower in PN-ALI and EMM	–	–	Higher MUC5AC in mAir cultures. PN-ALI, Epi and EMM cultures were negative for MUC5AC	–	–	
Leung et al. ^12^	No (BEBM –Lonza)	Comparison of AB-ALI vs PN-ALI	Bronchial (adult)	Thicker in PN-ALI	Lower in PN-ALI	Higher in PN-ALI	Similar in both media	–	–	Higher MUC5AC in PN-ALI	–	–
Broadbent et al. ^11^	No	Promocell vs PN-ALI media	Nasal (paediatric)	–	Similar in both media	Increased ciliated cells in PN-ALI but not significant	–	–	Smaller, more tightly packed cells in PN-ALI	Similar MUC5AC in both media	More RSV infected cells in PN-ALI	–
Saint-Criq et al. ^13^	Yes (F media)	Comparison of AB-ALI vs PN-ALI	Bronchial (adult)	Thicker in PN-ALI	Lower in PN-ALI	Higher in PN-ALI	–	Higher CFTR and baseline currents in PN-ALI	–	No difference in MUC5AC across both media	–	–
Ruiz García et al. ^10^	No (BEBM –Lonza)	Comparison of AB-ALI vs PN-ALI	Nasal (adult)	–	–	Higher in PN-ALI	–	–	–	No detection of MUC5AC or SCGB1A1 in AB-ALI	–	–
Lee et al. ^22^	Yes (F media)	Comparison of BEGM, PromoCell, LHC-8 and PN-ALI	Nasal (adult)	–	Lower in PN-ALI	Higher in BEGM	Lower in PN-ALI	–	–	Higher MUC5AC expression in PN-ALI	–	–

MEM, minimum essential media; USG, Ultroser-G (TM); PN-ALI, PneumaCult ALI; 50:50 BEBM and DMEM, AB-ALI; BEGM, bronchial epithelial growth medium; BEBM, bronchial epithelial cell growth basal medium; F media: CaCC, Calcium activated currents; AECGM, Promocell; CRC, conditionally reprogrammed culture # PN-Explus media to apical and basal side for initial 4 days on transwells; mAir, modified AECGM; Epi, MucilAir culture medium; EMM, EGM2-MucilAir-mixture; wt-CFTR, wild type CFTR; MUC5AC, Mucin 5AC.

Here, we compared the effects of two commonly used commercially available differentiation media in CRpBECs: PneumaCult and Bronchial Epithelial Growth Media (BEGM) based ALI media (Lonza). Media were compared in terms of their impact on morphological features, epithelial barrier integrity, ciliary activity, ion channel function, mucus production, viral replication kinetics, and antiviral responses (96 h post rhinovirus (RV)A1b infection) in CRpBECs. We found significant differences in morphological features and functional behavior (epithelial barrier function, ciliary activity, epithelial ion transport activity) between ALI cultures differentiated in PneumaCult and BEGM based media, but no significant difference in viral load or host antiviral responses after infection. These findings demonstrate how important the consideration of differentiation media is prior to the onset of culture-based experiments. This data highlights the impact that differing culture media have on overall CRpBECs morphology and emphasizes the importance of considering this when choosing the media for an individual study, and when comparing studies that have used different media.

## Materials and methods

### Human pBECs procurement and processing

The study was approved by the Hunter New England Area Health Service Ethics Committee (05/08/10/3.09) and the University of Newcastle (Newcastle, NSW, Australia) Safety Committee (R5/2017) and all methods were performed in accordance with relevant guidelines and regulations. Human pBECs were obtained by endobronchial brushing during fibre-optic bronchoscopy (as previously described^15^ from healthy subjects (*n* = 5) following written informed consent. All participants were non-smokers and had normal lung function with no evidence of respiratory disease in the preceding 4 weeks. Demographics of study participants are in Table 2.

**Table 2:** Clinical characteristics of healthy donors of primary broncho-epithelial cells.

Number, n	5
Age year (SD)	60 (9.5)
Male, n (%)	2 (40)
BMI (SD)	31.1 (7.3)
FEV1, % predicted (SD)	88.8(8.6)
FVC, % predicted (SD)	96.8 (11.6)
(FEV1/FVC) % (SD)	70.3 (4.1)

FEV1, forced expiratory volume in one second; FVC, forced vital capacity; SD, standard deviation; BMI, body mass index.

### CR cell expansion culture/co-culture

pBECs were previously established and expanded in standard BEGM Bulletkit (BEGM; Lonza). Passage 1 BEGM-expanded pBECs were subsequently added to collagen I coated flasks and co-cultured with an equal amount of irradiated NIH/3T3 feeder cells in F-media containing the ROCK inhibitor Y-27632 as described previously^5^ (Table S1), with and without weaning steps, as previously described^8^. For cells that did not undergo the weaning step, media changes were performed every alternate day until 80–90% confluence with complete F-media containing ROCK inhibitor. Cells were passaged by differential trypsinization using a Trypsin/EDTA reagent pack (Lonza). Viability and cell count were assessed using the trypan-blue method.

### NIH/3T3 feeder cell culture and irradiation

Cells of the NIH/3T3 mouse embryonic fibroblast line were cultured at 37 °C, with 5% CO_2_ in DMEM (Sigma D5796) supplemented with 10% FBS and 1% (v/v) penicillin/streptomycin (Life Technologies, Australia). Cells at 80–90% confluency were trypsinized, and pelleted cells were resuspended in fresh culture media. For generation of a fibroblast ‘feeder’ layer, the NIH/3T3 cell suspension was exposed to 30 Gy g-irradiation (RS 2000 X-Ray irradiator, RAD SOURCE) and then seeded into collagen I (PureCol;Advanced Biomatrix 5005) coated flasks at a 1:1 ratio with pBECs, as previously described^16^.

### ALI cultures

CR expanded pBECs were seeded on collagen I coated 24-well Transwell membranes (Corning, USA; 6.5 mm 0.4 µm pore polyester membrane Sigma CLS3470) at a density of 1.5 × 10^5^ cells/insert. Cells were then cultured in two different differentiation media, bronchial epithelial base medium and Dulbecco’s modified eagle medium BEBM:DMEM (50:50) and PneumaCult Ex Plus/ALI (STEMCELL Technologies). In BEBM:DMEM (50:50) differentiation, for the initial 24 h the cells were cultured in ALI initial media containing components listed in Table S2, for 3–5 days until confluent. Once confluent, apical media was removed (day 0 of ALI culture), basal media change was performed every second day with ALI final media that contains lower rhEGF concentrations (0.5 ng/mL), as previously described^17^. For PneumaCult differentiation, cells were cultured in PneumaCult Ex Plus for 4-5 days until 100% confluent. Cells were then cultured at ALI by removing the growth medium from the apical surface and replacing the basal medium with PneumaCult ALI medium supplemented with hydrocortisone and heparin (STEMCELL technologies) according to the manufacturer’s instructions (Table S3), as previously described^9^. In both conditions, basal media was changed every second day (with corresponding media type) until day 28 of ALI culture. The apical surface was washed with phosphate-buffered saline (PBS, no Ca^2+^ and Mg^2+^) at 37 °C once a week to remove excess mucus.

### Transepithelial electrical resistance (TEER) measurements

TEER was measured at 7-day intervals from 7 to 28 days in ALI cultures using an Epithelial Tissue Voltohmmeter (EVOM2) and STX2 electrodes (World Precision Instruments, Sarasota, USA). The STX2 electrodes were equilibrated in D-PBS prior to measurement. Pre-warmed D-PBS (Lonza) was added to the apical side of the insert 5 min prior to TEER measurement. Values were calculated after subtracting the blank value and according to the surface area of the inserts (0.33 cm^2^). TEER is expressed as Ω cm^2^. Results from each sample are the mean of 3–4 individual technical replicates.

### Transepithelial ion transport assay

Ussing chamber measurements were performed for cultures with resistance values > 200 Ω cm^2^. Differentiated HBE ALI cultures (28–30 days old) were mounted in circulating Ussing chambers (Physiologic Instruments VCC MC8 multichannel voltage/current clamp). The epithelium was voltage-clamped, and the resistance and short-circuit current (I_sc_) were measured. The resistance of a filter and Ringer solutions in the absence of cells was subtracted from all measurements. Three-five transwells were analyzed per donor per condition. For I_sc_ recordings cells were bathed in 5 ml of 37 °C Krebs-bicarbonate-Ringer containing (mM): 115 NaCl, 25 NaHCO_3_, 2.4 K_2_HPO_4_, 0.4 KH_2_PO_4_, 1.2 CaCl_2_, 1.2 MgCl_2_ and 10 glucose, pH 7.4. Ringer solutions were continuously gassed with 95% O_2_-5% CO_2_ and maintained at 37 °C. After recording the stable baseline I_sc_ for 15 min, cells were treated with pharmacological compounds (Table S4), in order: 100 µM amiloride, 10 µM forskolin and 100 µM IBMX, 30 µM CFTR_inh-172_ and 100 µM ATP. Data were collected and analyzed using Acquire and Analyze software (v2.3, Physiologic Instruments). Results from each sample are presented as the mean of 10–15 individual technical replicates.

### Cilia beating frequency and active area measurements

Cilia beating in differentiated CRpBECs cultures (frequency 3–20 Hz) was imaged on a Nikon eclipse Ti2 microscope (Nikon, Japan) and recorded with Video Savant 4.0 software using a high-speed digital video recorder. Recordings were made at 300 frames per second (fps) and a minimum of 512 frames were captured. Five fields of view were captured at random for each donor per differentiation media. Measurements of median cilia beat frequency were made using the CiliaFA plugin^18^. CiliaFA was used together with free open-source Fiji-ImageJ software (v1.53, ImageJ, US) and Microsoft Excel to perform analyses. Ciliary active area was calculated using Fiji-ImageJ software by performing built-in ‘Stack difference’ analysis to highlight areas of ciliary motion for each 512-frame file. Using the built-in Z-project plugin all 512 frames were ‘projected’ into 1 frame highlighting the regions of motile cilia. Thresholding was then applied identically to all projected images and active areas measured. Results from each sample are the mean of 5 fields of view.

### Alcian blue/periodic acid Schiff staining

ALI cultures at day 28 were washed with PBS, fixed in 10% neutral buffered formalin, embedded in paraffin and sectioned as previously described^17^. Alcian blue/periodic acid and Schiff (PAS) staining was performed also as previously described^17^. A minimum of 5 images were captured at random for each sample using a Nikon eclipse Ti2 microscope.

### Whole-mount immunolabelling and fluorescent microscopy

For immunolabelling of mucociliary differentiation markers, 28-day ALI cultures were first fixed in 4% paraformaldehyde for 15 min followed by storage in PBS containing 50 mM Glycine at 4 °C as previously described^17^. Whole mount membranes were permeabilized with 0.1% v/v Triton-X 100 (Sigma) and blocked with 10% v/v Goat serum (Genesearch, Aus) in PBS. Membranes were divided prior to antibody exposure. Antibodies against acetylated tubulin (T7451, Sigma) and ZO-1 (33-9100, Invitrogen) were incubated overnight at 4 °C. Membranes were washed in PBS and incubated with fluorescent secondary antibody Goat anti-mouse Alexafluor 594 (8890, Cell signaling technology, USA). Membranes were mounted on slides with Fluoromount-G containing DAPI (00-4959-52, Invitrogen, USA) placed under coverslips and sealed. A minimum of 3 images were captured per donor at random using Nikon Eclipse DS-Qi2 fitted with CoolLED box (pE-300). Images were then processed using ImageJ software (National Institute of Health, Bethesda, MD).

### Viral infection

pBEC cultures were inoculated apically with human RVA1b at an MOI of 0.1 for 2 h at 37˚C or media control. The apical inoculum was removed, and cultures were washed 2 times with PBS. Apical and basal media samples were collected at 24, 48, and 96 h post-inoculation and stored at -80 ˚C for cytometric bead array. Cells from the inserts were lysed with RLT buffer containing β-mercaptoethnaol (QIAGEN) for RNA.

### RNA extraction, cDNA synthesis, and quantitative PCR

Total RNA was extracted from ALI cultured cells lysed with RLT buffer containing β-mercaptoethnaol (QIAGEN) and frozen at -80˚C for downstream analysis. RNA was extracted using RNeasy Mini Kits (QIAGEN, Germany) according to the manufacturer’s instructions. RNA quality and quantity were measured using a nano-drop 2000 spectrophotometer (Thermo Scientific). RNA (200 ng) was reverse transcribed to cDNA using high-capacity cDNA reverse transcription kits (Thermo Scientific). qPCR was performed using a Quanstudio 6 as per the manufacturer’s instructions using TaqMan gene expression assays (ThermoFisher Scientific, Australia) and normalized to the ribosomal RNA 18 s) housekeeping gene (Table S5). Relative gene expression was calculated using the 2^−ΔΔCt^ (where Ct is the threshold cycle) method, as previously described^19–21^.

### Multiplex protein assessment

Apical supernatant from cells at each harvest time point was assessed using LEGENDPlex Human Anti-Virus Response Panel (BioLegend, San Diego, CA, USA), as per the manufacturer’s instructions and using a FACSCanto II flow cytometer (BD Biosciences, USA), as described previously^21^. Anti-viral interferons (IFN-β, λ1 and λ2/3) and inflammatory cytokines interleukins (IL-1β, 6), tumor necrosis factor-α (TNF-α), interferon (IFN)-γ-inducible protein (CXCL10/IP-10).

### Statistical analysis

Data for resistance measures, cilia activity and Ussing chamber analysis, which present the mean value for multiple technical replicates, are represented as dot plots with mean ± standard error of the mean (SEM). Data for all other measures are represented as with mean ± standard deviation (SD), with the respective number of experiments given in each figure legend. For statistical analysis, Wilcoxon matched-pairs signed rank test was used and data assessed with GraphPad Prism software (v9.3.1, San Diego, CA). A *p*-value < 0.05 was considered statistically significant.

## Results

### AB-ALI cultures have greater barrier function than PN-ALI cultures

**Figure 1:**
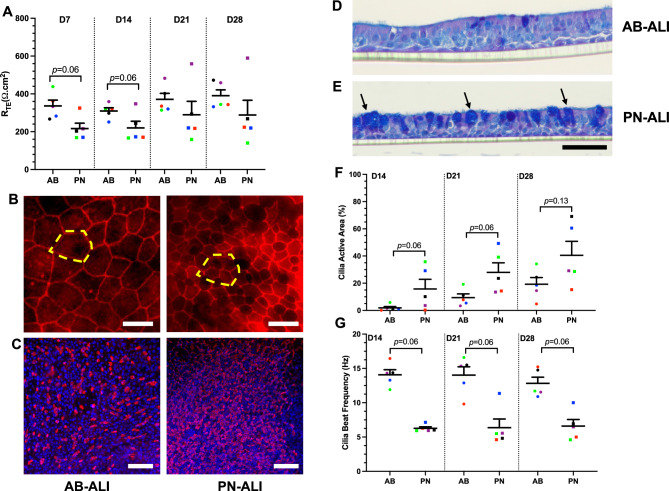
Structural and morphological characterization of AB-ALI and PN-ALI differentiated CRpBECs. (**A**) Trans-epithelial electrical resistance (TEER, R_TE_) values of AB-ALI and PN-ALI cultures. Each TEER data point represents an average of 10–15 transwells from each donor during the differentiation phase (d7-28). (**B**) Representative images of immunofluorescence staining of tight junctions (ZO-1, red). Yellow highlighted region shows 1 AB-ALI cell is equivalent to approximately 4 PN-ALI cells (Scale bar = 10 μm). (**C**) Representative images of immunofluorescence staining of ciliated cells (anti-acetylated α-tubulin (red)), and DAPI stained nucleated cells (blue) (Scale bar = 100 μm). (**D**, **E**) Representative images of Alcian blue/Periodic acid-Schiff-stained ALI cultures (Scale bar = 50 μm), black arrows indicate dense labelling for alcian blue. (**F**) Active cilia area and (**G**) cilia beat frequency measurements in AB-ALI and PN-ALI cultures. Five different fields of view were sampled per donor. A color-matched symbol in both groups represents an individual donor. n = 5. Data presented as mean ± SEM. Data were analyzed using a Wilcoxon matched-pairs signed rank teat, *p* < 0.05 was considered significant.

The effect of differentiation media on epithelial barrier integrity was assessed by measuring the TEER of the cultures. Resistance values varied among donors in both culture media, with a range of 190–800 Ω cm^2^. TEER data for both differentiation media displayed a general upward trend from day 7–28 post-ALI; with AB-ALI cultures increasing from 348 ± 12.45 to 388.1 ± 11.60 Ω cm^2^ and PN-ALI cultures increasing from 221.8 ± 6.92 to 301 ± 19.49 Ω cm^2^. At d7 and d14 time points AB-ALI had higher TEER than PN-ALI (*p* = 0.06). At d28 the TEER of AB-ALI cultures were higher though not significantly more than those of PN-ALI (388.1 ± 11.60 vs. 301 ± 19.49 Ω cm^2^, respectively) (Fig. 1A). These results suggest that differentiation media impacts epithelial barrier integrity, with AB-ALI cultures showing greater barrier function than PN-ALI cultures.

### PN-ALI differentiated CRpBECs are smaller and more ciliated than with AB-ALI media

Following CRpBECs differentiation, we observed several morphological differences between PN-ALI and AB-ALI cultures. Immunofluorescent localization of ZO-1 (Fig. 1B) revealed a considerable reduction in cell circumference/area when cultures were maintained in PN-ALI compared to AB-ALI. Indeed, these PN-ALI cells achieved only ~ 25% the area of their AB-ALI counterparts. Similar staining thickness and uniformity was observed for ZO-1 labelling between the two media. A much higher proportion of cells exhibited anti-acetylated tubulin fluorescent labelling in PN-ALI cultures compared to AB-ALI, reflecting an increase in the proportion of ciliated cells with the former (Fig. 1C). Longitudinal sections from each culture medium were cut and used for alcian blue periodic acid Schiff staining of mucus. Cells from cultures grown under PN-ALI conditions were variable in their overall mucin content, some matching closely to cultures grown under AB-ALI conditions while others had dense labelling for overlapping alcian blue, and PAS present in almost every ciliated-columnar cell. In contrast, AB-ALI cultures exhibited more consistent alcian blue/PAS staining that was present only in ~ 5–10% of columnar cells for all donor samples. Low PAS staining was detected outside of the overlap for both culture conditions (Fig. 1D, E).

### PN-ALI differentiated CRpBECs have elevated numbers of motile cilia but decreased cilia beat than AB-ALI cultures

The effect of differentiation media on ciliary function was assessed for two parameters: active ciliated area and, cilia beating frequency (CBF). Cilia motility was assessed on d14, 21, and 28 post-ALI, and beating cilia were observed from d14. PN-ALI cultures had significantly more ciliated cells with even coverage compared to AB-ALI, which showed sparsely distributed ciliated cells. The total active area of PN-ALI differentiated cultures were approximately double that of AB-ALI cultures at all time points (d14, 21 *p* = 0.06; d28; *p* = 0.13). The greatest difference in total active area was observed at d28 (40.52 ± 10.30 vs 19.27 ± 4.9%; *p* = 0.13) (Fig. 1F). The CBF data for both differentiation media exhibited stable values from day d14-28. When compared to PN-ALI, CBF values for AB-ALI were ~ two-fold higher (d14 14.06 ± 0.74 vs 6.26 ± 0.23 Hz; *p* = 0.06, Fig. 1G).

### PN-ALI differentiated CRpBECs have higher baseline and calcium-activated chloride currents than AB-ALI cultures

**Figure 2:**
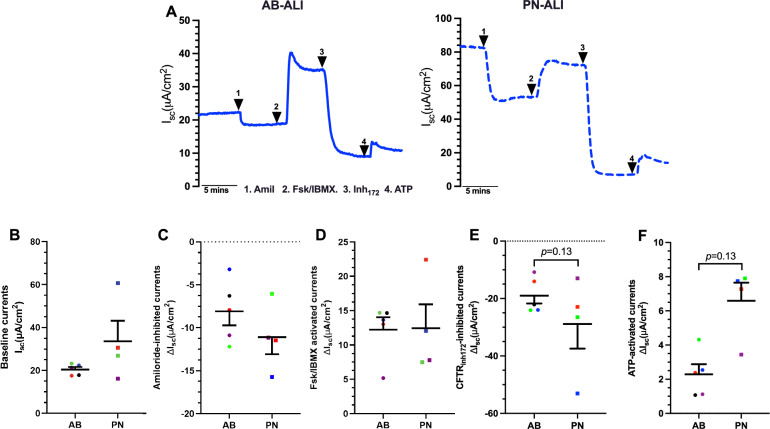
Ion transport measurements in AB-ALI and PN-ALI differentiated CRpBECs. (**A**) Representative Ussing chamber short circuit current (I_sc_) tracings from the same donor recorded at 37 °C for AB-ALI (blue line) and PN-ALI (dashed blue line) cultures. Mean values of (**B**) baseline short circuit currents (ΔI_sc_), (**C**) Amiloride inhibited ENaC currents, (**D**) Forskolin/IBMX activated CFTR, (**E**) CFTRinh172 inhibited CFTR and (**F**) ATP-activated CaCC currents. n = 5 for AB-ALI and n = 4 for PN-ALI. Data presented as mean ± SEM. A color-matched symbol in both groups represents an individual donor. Data were analyzed using a Wilcoxon matched-pairs signed rank test, *p* < 0.05 was considered significant.

The effect of differentiation media on ion transport was investigated by measuring transepithelial transport of amiloride-sensitive epithelial sodium channel (ENaC), forskolin-stimulated and CFTR_inh-172_-inhibited CFTR chloride secretion, and ATP-activated calcium-activated chloride channel (CaCC) currents (Fig. 2A). All ion channel measurements showed donor-to-donor variability (Table S6–7). Baseline currents were higher but not significantly so in PN-ALI than AB-ALI differentiated cultures (33.57 ± 9.54 vs 20.40 ± 1.17 µA/cm^2^; *p* = 0.0142, Fig. 2B). The inhibition of ENaC currents by amiloride appeared to be greater in PN-ALI than in AB-ALI cultures, though the difference was not statistically significant (ΔI_sc-Amiloride_ − 11.10 ± 1.98 vs − 8.11 ± 1.61 µA/cm^2^, Fig. 2C). CFTR mediated chloride secretion was assessed by cAMP agonist forskolin and a CFTR-specific inhibitor (CFTR_inh-_172) was used to ensure that the currents were mediated by the CFTR chloride channel, and it completely inhibited the forskolin-induced currents in both cultures. No difference in forskolin-induced CFTR currents was observed between AB-ALI and PN-ALI cultures (ΔI_sc-Fsk_ 12.23 ± 1.79 vs 12.43 ± 3.49 µA/cm^2^, Fig. 2D). Although the difference was not statistically significant, PN-ALI cultures tended to have higher CFTR_inh-_172 currents than AB-ALI cultures (ΔI_sc-CFTRinh-172_ − 28.87 ± 8.55 vs − 19.00 ± 2.76 µA/cm^2^, Fig. 2E). The addition of ATP at the end of the experiment resulted in transient CaCC currents. CaCC currents for PN-ALI were three times higher than AB-ALI cultures (ΔI_sc-ATP_, 6.59 ± 1.06 vs 2.29 ± 0.59, µA/cm^2^; *p* = 0.13, Fig. 2F). Overall, differentiation media affects ion transport with trends to differences in CFTR chloride and ENaC currents and large differences in CaCC currents.

### PN-ALI differentiated CRpBECs have increased FOXJ1 expression than AB-ALI cultures

**Figure 3:**
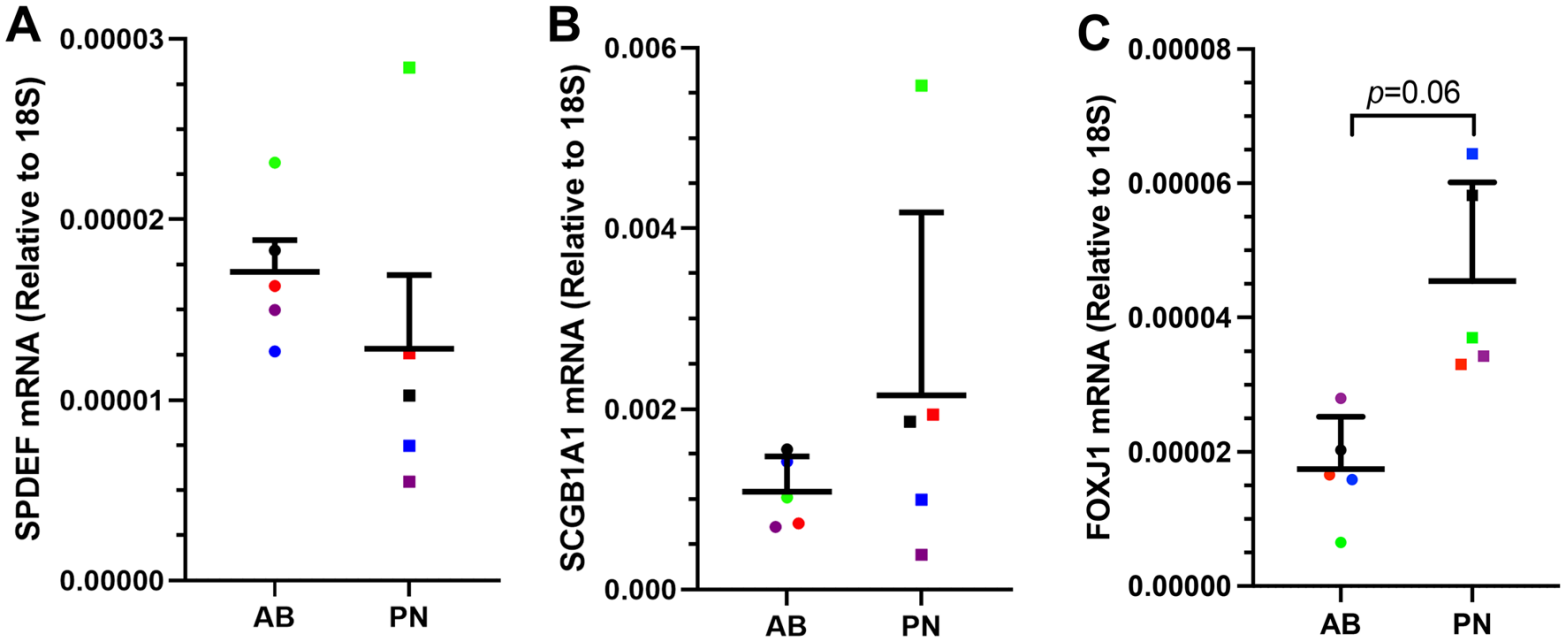
Cell marker gene expression in AB-ALI and PN-ALI differentiated CRpBECs. mRNA levels were measured by real-time quantitative PCR (RT-qPCR). Gene expression for (**A**) SPDEF, (**B**) SCGB1A1, (**C**) FOXJ1 is reported as [fold change (2^ddct)]. Data presented as mean ± SD. A color-matched symbol in both groups represents an individual donor. Data were analyzed using a Wilcoxon matched-pairs signed rank test, *p* < 0.05 was considered significant.

Cell markers were assessed by qPCR. There were no differences in the expression of the secretory cell markers SPDEF (Fig. 3A) enriched in goblet cells, or SCGB1A1 (Fig. 3B) enriched in club cells. However, expression of FOXJ1 (Fig. 3C), a marker of ciliogenesis, was increased in PN-ALI compared to AB-ALI cultures (*p* = 0.06).

### No difference in viral RNA and interferon protein secretion among the two PN-ALI and AB-ALI groups following RVA1b infection

**Figure 4:**
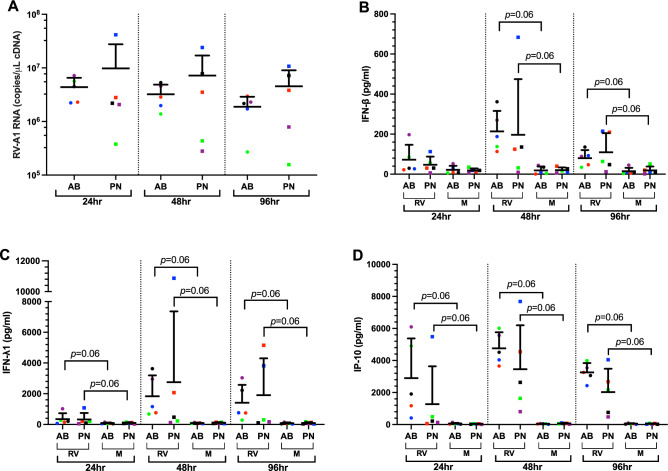
Viral load and cytokine production between AB-ALI and PN-ALI CRpBECs grown at air liquid interface and infected with rhinovirus RV-A1. (**A**) Viral RNA was quantified over time using real-time quantitative PCR (RT-qPCR). A multiplex protein quantification assay (LEGENDPlex) was performed using apical supernatant to determine changes in (**B**) IFN-β, (**C**) IFN-λ1, and (**D**) IP-10. Data presented as mean ± SD. A color-matched symbol in both groups represents an individual donor. Data were analyzed using a Wilcoxon matched-pairs signed rank test. *p* < 0.05 was considered significant.

Following RVA1b infection, viral replication kinetics (Fig. S1) were assessed by qPCR and anti-viral mediators in the apical supernatant by Multiplex at 24, 48 and 96 h, post infection. There was a variability in the viral RNA copy numbers in between individual donors in PN-ALI group at all the time points. The median viral RNA copy numbers were not statistically different among the groups at any time point (Fig. 4A). Production of IFN-β was induced by RVA1b infection at 48 and 96 h, post infection, (*p* = 0.06). (Fig. 4B). Similarly, IFN-λ and IP-10 appeared to be induced at all time points following RVA1b infection in both the groups, (Fig. 4C,D). Whilst there was a trend towards lower IP-10 production in PN-ALI after infection, there was no difference in interferon/cytokine abundance between PN-ALI and AB-ALI at any timepoint.

## Discussion

The airway epithelium is a critical barrier that plays a crucial role during infection and is often dysregulated in respiratory diseases such as asthma, chronic obstructive pulmonary disease (COPD), and cystic fibrosis (CF). Differentiated cultures of human pBECs are a valuable tool for investigating airway epithelial responses. However, only a limited number of pBECs can be obtained from human donors, with their capacity for expansion and proliferation restricted, making it challenging to study their function and response to interventions. The recent development of CR technology allows for large-scale expansion of pBECs, which can be used for larger and more complex studies. Combining the CR expansion method with ALI culture creates a powerful tool for the study of respiratory diseases, however, varying culture conditions including the choice of media may introduce variability in results. Several studies have compared a small number of parameters for different CR ALI culture media (Table 1) but have not compared differences in the effects of differentiation media. Here, we provide a detailed characterization of two widely used and well reported differentiation media for CR ALI cultures, characterizing a wide range of parameters to identify morphological, functional, and immune phenotypes. We show that PneumaCult and BEGM-based media result in differences in barrier function, active ciliated area/mucociliary function, cilia beating frequency, and ion transport. These differing effects need to be considered when reporting results, especially for assessments of morphology and studies of mucociliary clearance. In contrast, we found no differences in TEER and only minor differences in responses to infection with RVA1b in terms of viral load and antiviral responses.

The formation of tight junctions is crucial for the establishment of a functional epithelial barrier, and high TEER values are indicative of well-differentiated and tightly connected epithelium. Following differentiation, we found that both media yielded robust TEER values, suggesting that neither compromised epithelial tight junction formation. This is important for electrophysiological studies that rely on the integrity of the epithelial barrier. Interestingly, we observed that PN-ALI differentiated epithelia had a lower TEER compared to AB-ALI differentiated epithelia, consistent with previously published studies and likely attributable to a combination of reduced expression of sealing claudins and increased electrogenic ion transport^12,13,22,23^. Conversely, the higher TEER in AB-ALI differentiated cultures could be due to a squamous epithelium phenotype, which has been shown to confer higher TEER values^12,24^.

Significant differences in markers of mucociliary differentiation were observed between PN-ALI and AB-ALI most noticeably with the distribution of ciliated cells at the apical surface. PN-ALI cultures exhibited a relatively even covering of ciliated cells compared to the sparse distribution in AB-ALI. We also observed increased expression of FOXJ1 mRNA, a marker of ciliogenesis, in PN-ALI compared to AB-ALI cultures, which was consistent with prior findings^10–12^. We previously reported that CR of cells from asthmatic donors leads to lower expression of FOXJ1 and may suggest that differentiation in PN-ALI mitigates this effect^8^. Interestingly, although there was a greater area covered by cilia the CBF was lower in PN-ALI compared to AB-ALI cultures. This difference cannot be attributed to measurement techniques or inherent defects in cilia function in patients since individual healthy subjects can have cilia function below normal ranges^25,26^. Therefore, it is important to consider the likelihood of lower-than-normal cilia function when analyzing data using PN-ALI media. Given that the PN-ALI media was associated with a non-significant decrease in the goblet cell marker SPDEF and an increase in the club cell marker SCGB1A1, we speculate that differences in secretory function may contribute to more viscous mucus produced by PN-ALI differentiated cells, that may prevent cilia from beating as freely. However, prior studies by Ruiz Garcia et al.^10^, identified SCGB1A1 as being associated with goblet cells in PneumaCult ALI media. Further studies are needed to fully characterize secretory cell distribution and function and understand the underlying mechanisms of these differences.

For measurements of transepithelial ion transport using the Ussing chamber, PN-ALI cultures exhibited higher baseline currents most likely due to the small size and greater number of cells increasing the total surface area of the basolateral and apical membrane, as reported previously^24^. This is consistent with other studies which report that the lower resistance of monolayers cultured in PN-ALI is due to enhanced active ion transport as opposed to barrier dysfunction^12^. Moreover, PN-ALI cultures had higher though not significant differences in amiloride inhibited ENaC, this may be due to higher baseline levels of the function of these channels leading to an increase in overall ENaC conduction, as previously reported^27^. We did not observe any differences in CFTR conduction. We did also observe increased CaCC in PN-ALI cultures compared to AB-ALI. Although the mechanism is unclear, this may be due to increased electrogenic ion transport as suggested previously^13^ or higher expression of CaCC or more intracellular levels of Ca^2+^ in PN-ALI cultures.

Both media resulted in cultures that were successfully infected with RV with almost similar viral growth kinetics in line with the previous findings^11^. This and only other study compared the effect of differentiation media on viral growth kinetics or exogenous challenge such as infection. In agreement with the previous findings, we also observed higher though not significantly different levels of IFN-λ1 secreted from RV-infected PN-ALI cultures. Broadbent et al. proposed that this may be due to the higher cell density in the cultures^11^. The process of conditional reprogramming has been shown to impact viral load, growth kinetics, and immune responses but our findings suggest that the selection of differentiation media has little additional impact^8^. However, this should be validated for other differentiation media before mainstream use.

Our findings demonstrate that the choice of culture differentiation media can impact mucociliary differentiation, with alterations in epithelial ion channel function in CRpBECs, which is likely to result in important differences in these features when comparing results from different experiments. These observations have important implications for researchers working with in vitro models of the airway epithelium for disease modelling and highlight the need for a better understanding of the factors that influence mucociliary differentiation in ALI cultures. Furthermore, efforts should be made to establish standardized differentiation protocols for ALI cultures as well as to improve the accessibility of information regarding commercially available differentiation media.

## Supplementary Information

Supplementary Figures.

Supplementary Table 1.

Supplementary Table 2.

Supplementary Table 3.

Supplementary Table 4.

Supplementary Table 5.

Supplementary Table 6.

Supplementary Table 7.

## Data Availability

All data generated or analyzed during this study are included in the present article (and its Supplementary Information files).
