# Supplementary figures and images for: Comparison of commercially available differentiation media on cell morphology, function, and anti-viral responses in conditionally reprogrammed human bronchial epithelial cells
Source: Sci Rep. 2023 Jul 11;13:11200. doi: 10.1038/s41598-023-37828-0 (PMC10336057; doi:10.1038/s41598-023-37828-0)

Supplementary Figure 1

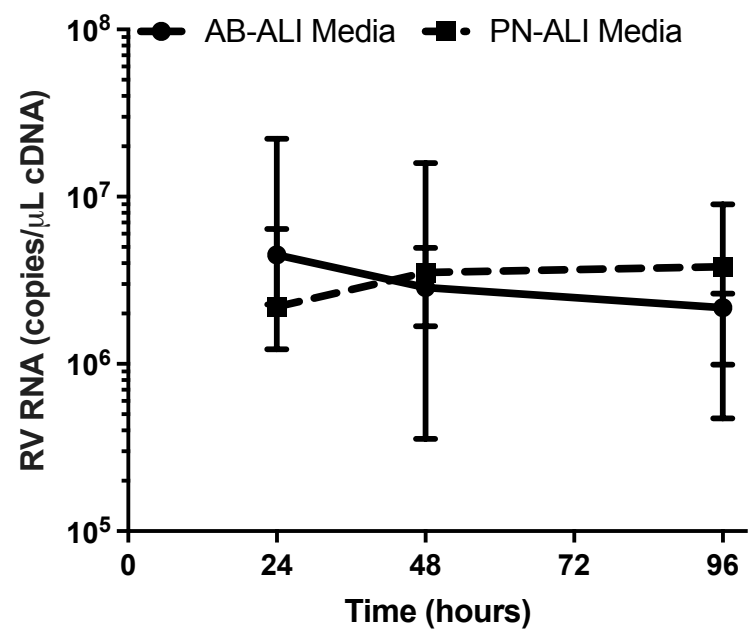

Figure S1: Line graph for viral growth kinetics in AB-ALI and PN-ALI CRpBECs.

Supplement: Supplementary file 1 — Supplementary Figures. [file 41598_2023_37828_MOESM1_ESM.pdf]
