# Supplementary material for: Comparison of commercially available differentiation media on cell morphology, function, and anti-viral responses in conditionally reprogrammed human bronchial epithelial cells
Source: Sci Rep. 2023 Jul 11;13:11200. doi: 10.1038/s41598-023-37828-0 (PMC10336057; doi:10.1038/s41598-023-37828-0)
Supplement: Supplementary file 2 — Supplementary Table 1. [file 41598_2023_37828_MOESM2_ESM.pdf]

Supplementary tables: Table S1. Components of CR cell expansion media

**Components**	**Concentration**	**Supplier**	**Catalogue number**
DMEM/Ham's F-12	67%	Sigma	Cat#51651C
DMEM, high glucose	33%	Sigma	Cat#D5796
Fetal bovine serum	5%(v/v)	Interpath	Cat#SFBSF15
Penicillin-Streptomycin	0.2%(v/v)	Life Tech	Cat#15070-063
Hydrocortisone	400ng/ml	Sigma	Cat#H0888
Insulin	5µg/ml	Sigma	Cat#I0516
Cholera toxin	8.4ng/ml	Sigma	Cat#C8052
hEGF	10ng/ml	Bioscientific	Cat#236-EG-200
Adenine	23.9µg/ml	Sigma	Cat#A2786
Y-27632	10µM	Enzo life sciences	Cat#ALX-270-333-M025
