# Supplementary material for: Comparison of commercially available differentiation media on cell morphology, function, and anti-viral responses in conditionally reprogrammed human bronchial epithelial cells
Source: Sci Rep. 2023 Jul 11;13:11200. doi: 10.1038/s41598-023-37828-0 (PMC10336057; doi:10.1038/s41598-023-37828-0)
Supplement: Supplementary file 3 — Supplementary Table 2. [file 41598_2023_37828_MOESM3_ESM.pdf]

Table S2. Components of Lonza ALI base differentiation media

Components	Concentration	Supplier
BEBM	50%	Lonza, Cat#CC3171
DMEM	50%	Sigma, Cat#D5796
Hydrocortisone	0.1%	Lonza, Cat# (CC- 4031 F)
Insulin, Bovine	0.1%	Lonza, Cat# (CC – 4021 F)
Epinephrine	0.1%	Lonza, Cat# (CC – 4221 F)
Bovine Pituitary Extract	0.4%	Lonza, Cat# (CC – 4009F)
Transferrin	0.1%	Lonza, Cat# (CC – 4205F)
Ethanolamine	80µM	Sigma, Cat#E0135
hEGF	10 ng/ml	Bioscientific Cat#236-EG-200
All-trans retinoic acid	30ng/ml	Sigma, Cat#R2625
$MgCl_2$	0.3mM	Sigma, Cat#M8266
$MgSO_4$	0.4mM	Sigma, Cat#M2643
BSA	0.5mg/ml	Sigma, Cat#A8806
Penicillin/streptomycin	2%	Life Tech, Cat#15070-063
Amphotericin B solution	250µg/ml	Sigma, Cat#A2942
