# Supplementary material for: Comparison of commercially available differentiation media on cell morphology, function, and anti-viral responses in conditionally reprogrammed human bronchial epithelial cells
Source: Sci Rep. 2023 Jul 11;13:11200. doi: 10.1038/s41598-023-37828-0 (PMC10336057; doi:10.1038/s41598-023-37828-0)
Supplement: Supplementary file 4 — Supplementary Table 3. [file 41598_2023_37828_MOESM4_ESM.pdf]

Table S3. Components of StemCell media expansion and differentiation

**Components**	**Concentration**	**Supplier**
PneumaCult^{TM} Ex-Plus media		StemCell, Cat#05041
PneumaCult^{TM} Ex-Plus media 50x supplement	Unknown	StemCell, Cat#05042
PneumCult^{TM} ALI maintenance media		StemCell, Cat#05002
PneumaCult^{TM}-ALI 10x supplement	Unknown	StemCell, Cat#05003
PneumaCult^{TM}-ALI maintenance supplement 100x	Unknown	StemCell, Cat#05006
Hydrocortisone		StemCell, Cat#07925
Heparin		StemCell, Cat#07980
Penicillin/streptomycin	2%	Life Tech, Cat#15070-063
Amphotericin B solution	250µg/ml	Sigma, Cat#A2942
