# Supplementary material for: Comparison of commercially available differentiation media on cell morphology, function, and anti-viral responses in conditionally reprogrammed human bronchial epithelial cells
Source: Sci Rep. 2023 Jul 11;13:11200. doi: 10.1038/s41598-023-37828-0 (PMC10336057; doi:10.1038/s41598-023-37828-0)
Supplement: Supplementary file 5 — Supplementary Table 4. [file 41598_2023_37828_MOESM5_ESM.pdf]

Table S4. Pharmacologic compounds used for Ussing chamber measurements

	**Site of addition (Apical/Basolateral)**	**Pharmacological activity**	**Supplier**	**Catalogue number**
Amiloride	A	Inhibit Epithelial sodium channels (ENaC)	Sigma-Aldrich	Cat#A7410
Forskolin	A and B	cAMP activator (activate CFTR)	Sigma-Aldrich	Cat#F6886
IBMX (3-Isobutyl-1-methylxanthine)	A and B	Phosphodiesterase inhibitor	Sigma-Aldrich	Cat#I5879
CFTRInh-$_{172}$	A	CFTR inhibitor	Selleckchem	Cat#S7139
ATP	A	Calcium activated chloride channel activator (CaCC)	Sigma-Aldrich	Cat#A2383
