# Supplementary material for: Comparison of commercially available differentiation media on cell morphology, function, and anti-viral responses in conditionally reprogrammed human bronchial epithelial cells
Source: Sci Rep. 2023 Jul 11;13:11200. doi: 10.1038/s41598-023-37828-0 (PMC10336057; doi:10.1038/s41598-023-37828-0)
Supplement: Supplementary file 6 — Supplementary Table 5. [file 41598_2023_37828_MOESM6_ESM.pdf]

Table S5. Genes assessed by PCR

Gene	TaqMan Assay ID	Dye-Probe	Supplier	Catalogue number
FOXJ1	Hs00201755_m1	FAM-MGB	ThermoFisher Scientific	4331182
SPDEF	Hs0017942_m1	FAM-MGB	ThermoFisher Scientific	4331182
SCGB1	Hs00171092_m1	FAM-MGB	ThermoFisher Scientific	4331182
18s	N/A	VIC-MGB	ThermoFisher Scientific	4319413E
