# Supplementary material for: Comparison of commercially available differentiation media on cell morphology, function, and anti-viral responses in conditionally reprogrammed human bronchial epithelial cells
Source: Sci Rep. 2023 Jul 11;13:11200. doi: 10.1038/s41598-023-37828-0 (PMC10336057; doi:10.1038/s41598-023-37828-0)
Supplement: Supplementary file 7 — Supplementary Table 6. [file 41598_2023_37828_MOESM7_ESM.pdf]

**Table S6. Short-circuit currents and electrophysiologic parameters in ALI base differentiation media.** Data represent short circuit current values for baseline currents and resistance of monolayers. Amiloride inhibited ENaC currents (ΔAmil), Forskolin/IBMX stimulated cAMP currents, $CFTR_{Inh}$-172 inhibited currents **(**ΔFsk + $CFTR_{Inh}$-172**),** and ATP-activated currents (ΔATP). Values represented are (mean ± SEM); D = Donor.

| **AB-ALI** | **Baseline currents (µA/cm²)** | **Resistance (Ω.cm²)** | **ΔAmil (µA/cm²)** | **ΔFsk/IBMX (µA/cm²)** | **ΔFsk/IBMX + $CFTR_{inh}$172 (µA/cm²)** | **ΔATP (µA/cm²)** |
|---|---|---|---|---|---|---|
| D1 | 17.76 ± 1.29 | 319.4 ± 25.99 | -6.29 ± 0.50 | 14.63 ± 2.28 | -22.11 ± 1.77 | 1.07 ± 0.35 |
| D2 | 22.30 ± 0.81 | 227.7 ± 14.24 | -3.20 ± 0.64 | 13.63 ± 2.02 | -23.97 ± 1.89 | 2.54 ± 0.27 |
| D3 | 23.20 ± 1.129 | 217.3 ± 25.47 | -12.21 ± 1.27 | 14.68 ± 0.89 | -24.07 ± 0.80 | 4.32 ± 0.30 |
| D4 | 17.50 ± 3.012 | 164.5 ± 29.21 | -7.93 ± 0.19 | 13.01±1.67 | -14.03 ± 1.48 | 2.38 ± 0.30 |
| D5 | 21.23 ± 2.03 | 140.5 ± 20.99 | -10.90 ± 0.34 | 5.17 ± 1.22 | -10.80 ± 0.50 | 1.12 ± 0.15 |
