# Supplementary material for: Comparison of commercially available differentiation media on cell morphology, function, and anti-viral responses in conditionally reprogrammed human bronchial epithelial cells
Source: Sci Rep. 2023 Jul 11;13:11200. doi: 10.1038/s41598-023-37828-0 (PMC10336057; doi:10.1038/s41598-023-37828-0)
Supplement: Supplementary file 8 — Supplementary Table 7. [file 41598_2023_37828_MOESM8_ESM.pdf]

Table S7. Short-circuit currents and electrophysiologic parameters in PneumaCult differentiation media. Data represent short circuit current values for baseline currents and resistance of monolayers. Amiloride inhibited ENaC currents (ΔAmil), Forskolin/IBMX stimulated cAMP currents, $CFTR_{Inh}$-172 inhibited currents **(**ΔFsk + $CFTR_{Inh}$-172**),** and ATP-activated currents (ΔATP). Values represented are (mean ± SEM); D = Donor.

**PN ALI**	**Baseline currents (µA/cm^2)**	**Resistance (Ω.cm^2)**	**ΔAmil (µA/cm^2)**	**ΔFsk/IBMX (µA/cm^2)**	**ΔFsk/IBMX + $CFTR_{inh}$172 (µA/cm^2)**	**ΔATP (µA/cm^2)**
D1	-	-	-	-	-	-
D2	60.67 ± 11.23	88.77 ± 8.24	-15.73 ± 7.46	12.07 ± 3.95	-53.05 ± 6.48	7.74 ± 0.89
D3	26.88 ± 3.84	69.10 ± 5.29	-6.05 ± 1.26	7.47 ± 0.69	-26.52 ± 4.43	7.90 ± 2.51
D4	30.63 ± 1.018	117.6 ± 10.63	-11.48 ± 0.68	22.40 ± 1.93	-22.93 ± 2.84	7.28 ±0.70
D5	16.10 ± 0.71	211.0 ± 28.29	-11.15 ± 0.74	7.78 ± 1.19	-12.99 ± 1.20	3.44 ± 0.55
